# Regenerative stem cell therapy for stroke in Europe (RESSTORE): a multicenter randomized controlled efficacy clinical trial

**DOI:** 10.3389/fstro.2024.1416490

**Published:** 2024-09-27

**Authors:** Loïc Legris, Anaick Moisan, Assia Jaillard, Louise Bonnet, Thierry Moulin, Igor Sibon, Emmanuel Touzé, Isabelle Favre-Wiki, Charlotte Cordonnier, Lucie Dellaschiava, Mikael Mazighi, Charlotte Rosso, Sonia Alamowitch, David Calvet, Marianne Barbieux-Guillot, Stephan Roux, Alain-Ali Mojallal, Fabien Boucher, Antoine Thuriot, Julie Soulard, Bernadette Naegele, Dominic Perennou, Matthieu Roustit, Zaza Putkaradze, Marc Hommel, Audrey Lehmann, Julien Colombat, Fatima Chorfa, Delphine Maucort-Boulch, Laurent Lamalle, Sylvie Grand, Alexandre Krainik, Olivier Detante

**Affiliations:** ^1^Univ. Grenoble Alpes, Inserm, U1216, Stroke Unit, Department of Neurology, CHU Grenoble Alpes, Grenoble Institut Neurosciences, Grenoble, France; ^2^Cell Therapy and Engineering Unit, EFS Rhône Alpes, Saint Ismier, France; ^3^Univ. Grenoble Alpes, AGEIS EA 7407, Research 3T-MRI, IRMaGe, CHU Grenoble Alpes, Grenoble, France; ^4^University of Franche-Comté, UFR Santé, Department of Neurology, CHU Besançon, Besançon, France; ^5^Department of Neurology, Stroke Unit, CHU Bordeaux, Bordeaux, France; ^6^Department of Neurology, Stroke Unit, CHU Caen, Medical University of Caen, Caen, France; ^7^Univ. Grenoble Alpes, Department of Neurology, Stroke Unit, CHU Grenoble Alpes, Grenoble, France; ^8^Inserm, CHU Lille, U1172 Lille Neuroscience and Cognition (LiINCog), University of Lille, Lille, France; ^9^Department of Neurology, Stroke Unit, CHU Lariboisière, APHP Nord, Interventional Neuroradiology Department and Biological Resources Center, Rothschild Foundation Hospital, FHU Neurovasc, INSERM 1144, University of Paris City, Paris, France; ^10^Inserm U 1127, CNRS UMR 7225, Sorbonne Université, UPMC Univ Paris 06 UMR S 1127, Institut du Cerveau et de la Moelle épinière, ICM, APHP Stroke Unit, Pitié Salpêtrière Hospital, Paris, France; ^11^AP-HP, Urgences Cérébro-Vasculaires, Hôpital Pitié-Salpêtrière, Sorbonne Université, Paris, France; ^12^STARE Team, iCRIN, Institut du Cerveau, INSERM U1266, Paris, France; ^13^Department of Neurology, Stroke Unit, GHU-Paris Psychiatry and Neurosciences, Sainte Anne Hospital, Institute of Psychiatry and Neuroscience of Paris (IPNP), INSERM, U1266, University of Paris City, Paris, France; ^14^Department of Neurology, Stroke Unit, CHU Toulouse, Toulouse, France; ^15^Cell Therapy and Engineering Unit, EFS Bourgogne Franche Comté, Besançon, France; ^16^Department of Plastic Surgery, Croix Rousse Hospital, HCL, Lyon, France; ^17^Univ. Grenoble Alpes, Department of Neurorehabilitation, CHU Grenoble Alpes, LPNC, Grenoble, France; ^18^Univ. Grenoble Alpes, Inserm, Clinical Investigation Center (CIC), CHU Grenoble Alpes, Grenoble, France; ^19^Univ. Grenoble Alpes, AGEIS EA 7407, Grenoble, France; ^20^Univ. Grenoble Alpes, Department of Pharmacy, CHU Grenoble Alpes, Grenoble, France; ^21^Department of Research and Innovation, CHU Grenoble Alpes, Grenoble, France; ^22^Hospices Civils de Lyon, Pôle Santé Publique, Service de Biostatistique et Bioinformatique, Lyon, France; ^23^Université Lyon 1, Villeurbanne, France; ^24^CNRS, UMR 5558, Laboratoire de Biométrie et Biologie Evolutive, Equipe Biostatistique-Santé, Villeurbanne, France; ^25^Univ. Grenoble Alpes, Inserm, CHU Grenoble Alpes, CNRS, IRMaGe, 38000, Grenoble, France; ^26^Univ. Grenoble Alpes, INSERM, U1216, Department of Neuroradiology, CHU Grenoble Alpes, Grenoble Institut Neurosciences, Grenoble, France; ^27^Univ. Grenoble Alpes, INSERM, U1216, Research 3T-MRI, IRMaGe, Department of Neuroradiology, CHU Grenoble Alpes, Grenoble Institut Neurosciences, Grenoble, France

**Keywords:** stroke, cell therapy, stem cells, ADSC, regeneration, recovery, RCT

## Abstract

**Introduction:**

Encouraging the activation of brain repair mechanisms and fostering spontaneous functional recovery in stroke patients hold great promise for alleviating the global burden of this condition and allowing an extended therapeutic time window. Cell-based regenerative therapy (with mesenchymal stem/stromal cells, such as adipose-derived stem cells [ADSCs]) is particularly attractive considering its excellent safety profile, low immunogenicity after allogeneic application, and well-established functional benefits on stroke recovery in animal models. This study aims to assess the efficacy and safety effects of intravenous (IV) infusion of freshly cultured allogeneic ADSCs on recovery after ischemic stroke.

**Population and methods:**

RESSTORE is a multicentric, randomized 1:1 controlled double-blind clinical trial. Eighty patients will be enrolled in nine French stroke centers. The main inclusion criteria are ≥18 years of age, acute hemispheric ischemic stroke, and a National Institutes of Health Stroke Scale (NIHSS) score of ≥7, including a motor subscore of ≥3. According to the previous dose-escalation safety trial data, the maximum tolerated dose of 3 million ADSCs/kg was selected. IV infusion was performed within 10 days following stroke onset, with a follow-up over 2 years.

**Outcomes:**

The primary endpoint is the motor NIHSS subscore, computed as the sum of the upper limb, lower limb, and hand scores, measured 6 months after stroke onset to assess motor recovery. The secondary outcomes are the occurrence of death/serious adverse events, clinical scores (the detailed NIHSS scores, Montreal Cognitive Assessment scores, modified Rankin Scale scores, Aphasia Handicap Scores, Depression Intensity Scale Circles scores, Fatigue Scale scores, etc.), immune monitoring (for the first 30 patients), and multimodal biomarkers derived from diffusion and functional magnetic resonance imaging.

**Discussion:**

This study may provide some evidence for the effects of freshly cultured allogenic ADSCs IV infusion in subacute stroke that may help design a larger international randomized controlled trial.

**Clinical trial registration:**

https://clinicaltrials.gov/, identifier: NCT03570450.

## 1 Introduction

In the European Union, approximately 6 million people are impacted by stroke, with 1.1 million new cases reported each year. Despite experiencing some degree of spontaneous recovery, more than 60% of stroke survivors contend with lasting impairments, resulting in significant burdens for both patients and their families, with broader societal implications. The stroke burden is expected to increase due to the aging population, the sharp rise in diabetes, and obesity reaching a pandemic level (GBD 2019 Stroke Collaborators, 2021).

Current treatment options are limited in the acute phase to intravenous (IV) thrombolysis, mechanical thrombectomy, aspirin within 48 h, decompressive craniectomy for large strokes, a stroke care network for intensive care management, and neurorehabilitation. After experiencing a stroke, the majority of survivors still endure sensorimotor and cognitive disabilities, amplifying the stroke burden on rehabilitative care. Hence, the demand for treatments that extend beyond prevention and acute care to be effective is urgent. However, developing novel therapies requires a sophisticated understanding of stroke pathophysiology. It is well known that stroke damages not only neurons but also involves both brain cells and the surrounding extracellular matrix in a “glio-neurovascular niche” that interacts with the peripheral immune system (Detante et al., 2023). For these reasons, new therapies should target all these systems to perhaps avoid the failures of past clinical translational attempts to develop specific protective drugs (Dirnagl and Endres, 2014).

A promising approach involves activating brain repair mechanisms and fostering spontaneous functional recovery using regenerative therapies. A major advantage is the extended therapeutic window of up to days or months after stroke, making this treatment available to a much larger number of stroke patients. Cell-based regenerative therapies have emerged as attractive approaches for stroke (Detante et al., 2023; Boncoraglio et al., 2019). Various cell types and strategies have demonstrated significant improvement in experimental studies. Of particular interest are mesenchymal stem/stromal cells (MSCs), which can be easily derived from multiple sources, including adipose tissue (adipose-derived stem cells, ADSC). In addition, their excellent safety profile and low immunogenicity after allogeneic application may enable their use as an “off-the-shelf” therapeutic product (Toyserkani et al., 2017). Concerning the delivery route, IV cell infusion, a non-invasive, and safe method that provides a broad distribution of cells close to ischemic tissue, has immediate access to clinical applications.

Although a prior meta-analysis hinted at the potential benefits of cell therapy for stroke patients (Detante et al., 2017), individual clinical trials have yet to yield significant results (Hess et al., 2017; Moniche et al., 2023; Houkin et al., 2024). Several factors have been suggested, including the cell type and the timing of cell administration after a stroke, which may be influenced by the potential delay in *in vitro* amplification. Additionally, the targeted mechanisms of action—whether focusing on acute brain protection, delayed brain repair, trophic systemic transient effects, or graft survival and integration—could also contribute to the lack of significant results. Moreover, using freshly cultured stem cells instead of frozen stem cells can lead to better therapeutic outcomes by ensuring higher cell viability and functionality.

Utilizing global outcome measures (e.g., modified Rankin Scale [mRS], Barthel Index, and the EuroQOL) could contribute to the observed limited efficacy (Hess et al., 2017; Houkin et al., 2024). Intriguingly, although motor performance is frequently assessed in experimental studies to evaluate the effects of cell therapy, it is not commonly examined in clinical randomized controlled trials (RCTs). According to the results of a previous study (Jaillard et al., 2020), we hypothesized that quantitative motor behavior and functional magnetic resonance imaging (MRI) measurements may provide objective and accurate measures of outcomes, resulting in more sensitive detection of treatment effects.

Therefore, our aim was to design an RCT to assess the effects of freshly cultured ADSCs in patients with subacute stroke.

The optimal window after stroke for cell administration remains a debate. Because the expected trophic support is the main mechanism of MSC injections occurring days to weeks after stroke onset and considering the delay required for the production and delivery of freshly cultured cells (5–7 days), we targeted the 7–10 days following stroke onset to administer IV ADSCs in the RESSTORE clinical trial.

The RESSTORE clinical trial includes two phases. The first phase, 1a, a first-in-human trial, was a dose escalation safety study including 17 patients with an acute first-ever ischemic stroke to determine the highest well-tolerated, safe single IV dose of 1–3 million ADSC/kg administered 7–10 days after stroke onset. The RESSTORE 1a study was completed in 2022, showing no cell-related adverse events for all treatment doses (preliminary data) (Detante et al., 2022). Therefore, the highest dose of 3 million ADSC/kg was selected for the second phase of the study, RESSTORE 1b.

RESSTORE 1b, a RCT, started in October 2023. The primary objective is to assess the efficacy of IV 3 million ADSC/kg on motor recovery 6 months following stroke. The secondary objectives are to assess ADSC safety and efficacy using neurological and physiotherapy clinical scores and biological and multimodal MRI parameters.

## 2 Methods and analysis

### 2.1 Study design

RESSTORE 1b is a double-blind single-dose multicenter prospective RCT.

**Figure 1:**
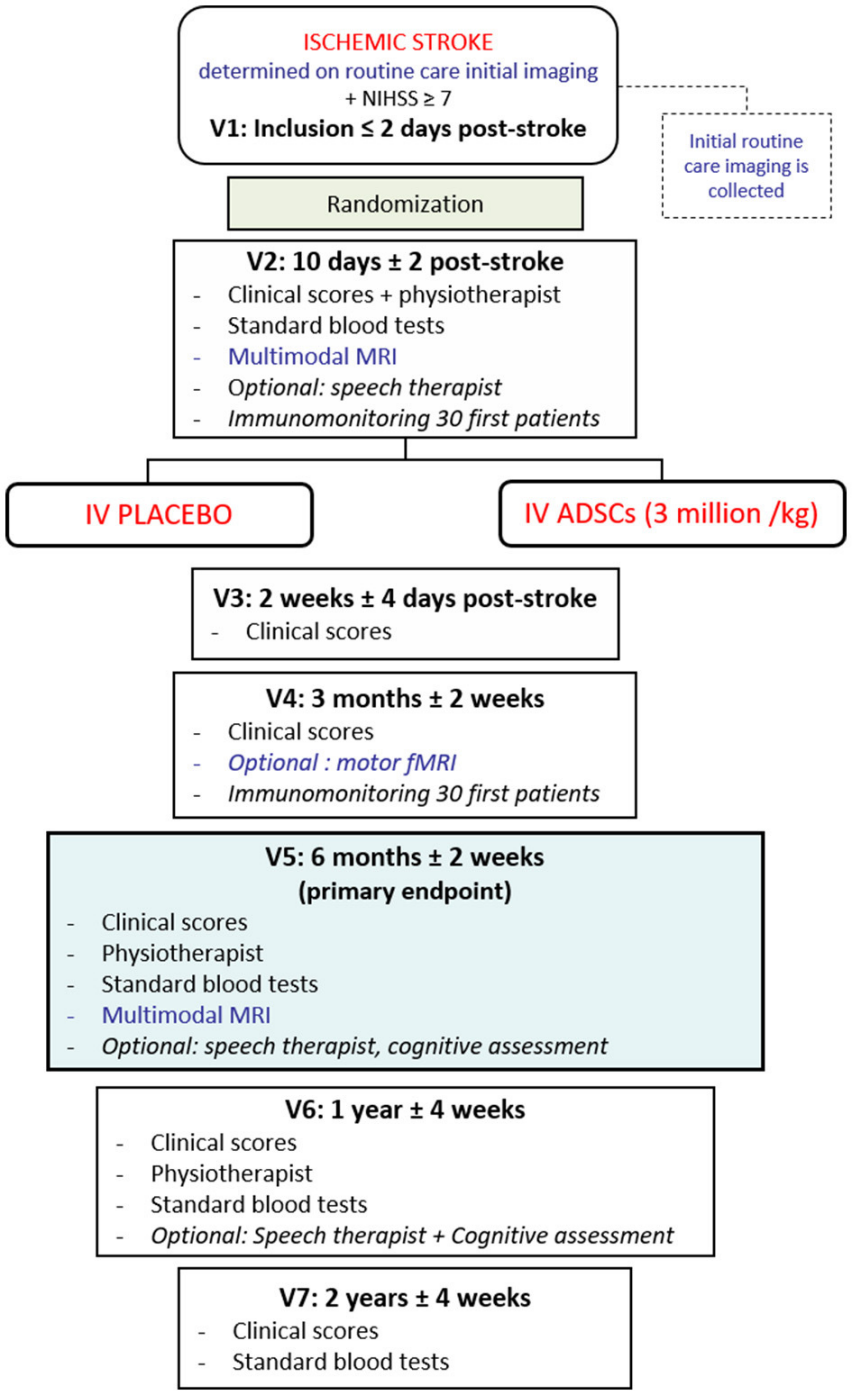
Experimental flowchart of RESSTORE trial. ADSCs, adipose-derived stem cells; IV, intravenous; fMRI, functional magnetic resonance imaging; MRI, magnetic resonance imaging; NIHSS, National Institute of Health Stroke Scale; V^*^, visit number ^*^.

The study flowchart is presented in Figure 1.

For each patient randomized in RESSTORE trial, seven visits are planned, from the inclusion (Visit 1) to the 2-year follow-up (Visit 7). The primary endpoint will be evaluated at 6 months (motor sub-score of the NIHSS). Safety and efficacy will be assessed by clinical scores and biological and multimodal MRI markers.

### 2.2 Patient population, inclusion, and non-inclusion criteria

This study will recruit 80 patients from 9 stroke comprehensive centers in France. The inclusion criteria are as follows: age ≥18 years, hemispheric ischemic stroke (>1.5 cm on 2 imaging slices, as determined by the first routine care brain imaging following stroke onset, either computed tomography scanner or diffusion-weighted MRI), no previous handicap, and the ability to follow a neurorehabilitation program. To assess ADSC efficacy, we target patients with moderate to severe stroke. Thus, eligible patients have a National Institute of Health Stroke Scale (NIHSS) score ≥7, with a motor sub-score (upper, lower limbs, and hand) ≥3, and no planned or performed decompressive craniectomy. IV thrombolysis and/or mechanical thrombectomy can be performed based on international guidelines.

All inclusion and non-inclusion criteria are listed in Table 1.

**Table 1:** Inclusion and non-inclusion criteria.

**Inclusion criteria**
Subjects will be eligible for the study if they meet all the following criteria: 1. Male or female >18 years old. 2. Hemispheric ischemic stroke (>1.5 cm on 2 imaging slices, determined on the first routine care imaging, i.e., CT scanner or diffusion-weighted MRI) admitted to the stroke unit within the first 24 h after stroke onset. 3. Patient must be included within the first or second day after stroke onset (signature of informed consent and randomization; i.e., between 24 h and 48 h from stroke onset) and must be able to receive investigation treatment within the first 10 days. 4. NIHSS score ≥7, including motor score (upper and lower limbs and hands) ≥3. 5. No decompressive craniectomy procedure (planned or performed). 6. Patient able to follow a rehabilitation program. 7. Modified Rankin Scale = 0 before stroke onset. 8. Obtained signed informed consent from the patient or legally acceptable representative. 9. Negative pregnancy test for women of childbearing age.
**Non-inclusion criteria**
Subjects will not be eligible for the study if they meet any of the following: 1. Contra-indication for MRI (*contra-indication to gadolinium is NOT a non-inclusion criterion but it is a contra-indication to optional injected MRI*). 2. Coma (score of 2 or more on item 1a of the NIHSS related to awareness). 3. Evidence on neuroimaging (CT or MRI) of a brain tumor, cerebral edema with midline shift and a clinically significant compression of ventricles, cerebellar or brainstem infarction, or subarachnoid hemorrhage, or intracerebral parenchymal hematoma (*petechial small hemorrhages, defined as Heidelberg Bleeding Classification grade HI-1and HI-2, are NOT a non-inclusion criteria*). 4. Severe leukoaraiosis (Fazekas scale = 3 for periventricular lesions). 5. Previous documented stroke. 6. Active endocarditis, pneumonia, AIDS, active hepatic disease due to HBV, or HCV (*a controlled infection is NOT a non-inclusion criterion*). 7. Active inflammatory and/or autoimmune diseases (such as Crohn's disease, lupus, rheumatoid polyarthritis, renal, or liver immune pathology). 8. History of cancer. 9. Preexisting dementia. 10. A health status, any clinical condition (e.g., short life expectancy or coexisting disease), or other characteristic that precludes appropriate diagnosis, treatment, or follow-up in the trial. 11. Surgical or endovascular procedure planned in the following 3 months. 12. Pregnancy/breastfeeding (women of childbearing age should have a negative pregnancy test prior to inclusion). 13. Patients who are participating in another therapeutic trial or who have previously participated in a biotherapy trial. 14. Non-membership to a social security scheme. 15. Inability or unwillingness of the individual or their legal guardian/representative to provide written informed consent, according to national regulations. 16. Person deprived of liberty by judicial order 17. Person under guardianship or curatorship.

CT, computed tomography; MRI, magnetic resonance imaging; NIHSS, National Institutes of Health Stroke Scale.

### 2.3 Treatment and intervention

According to the advice from the adjudication committee and the Data Safety Monitoring Board (DSMB) about the data from the dose-escalation previous study (RESSTORE phase 1a), the maximum tolerated dose of 3 million ADSC/kg has been selected.

**Figure 2:**
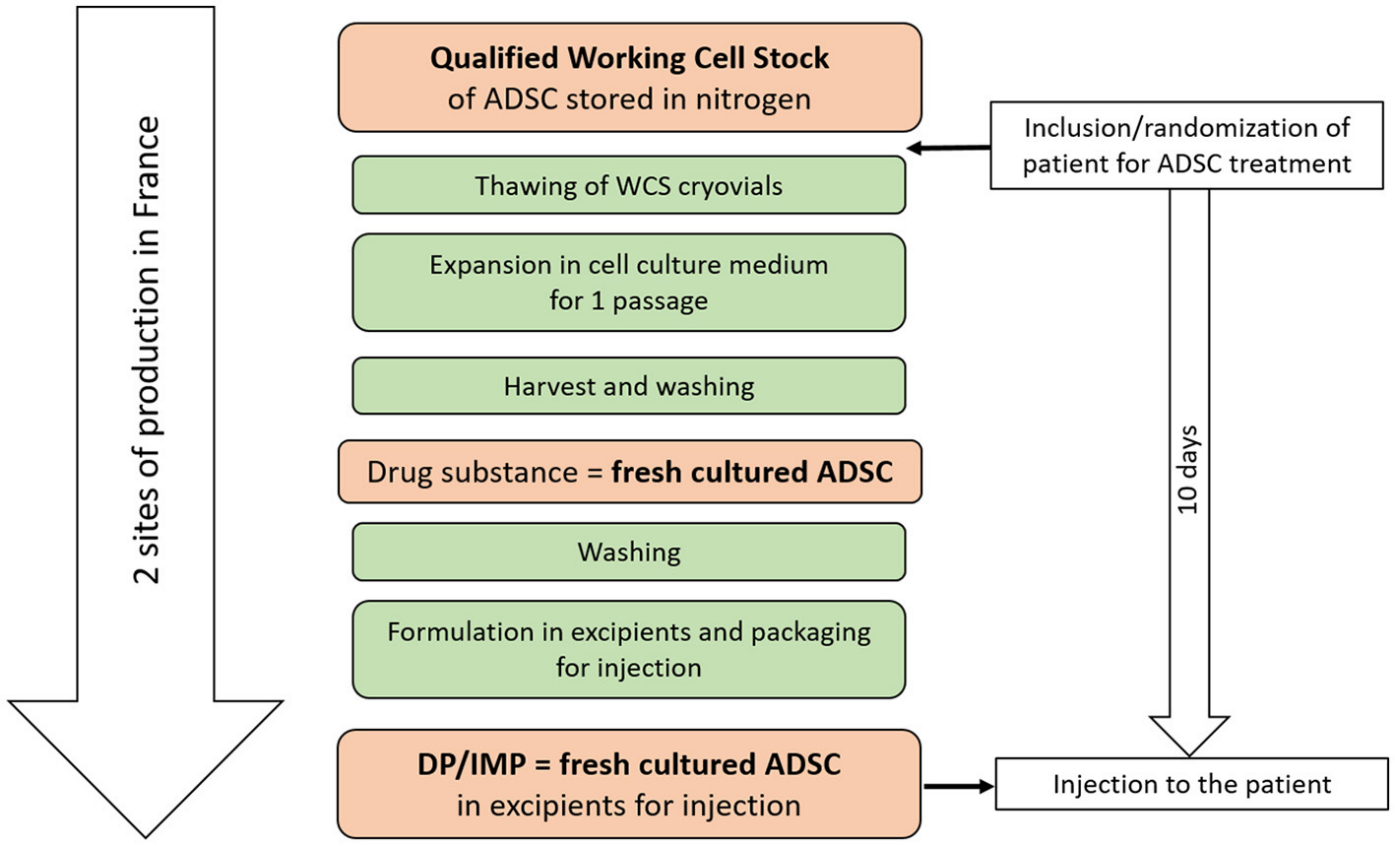
Flowchart of adipose-derived stem cells (ADSCs) production. DP/IMP, drug product/investigational medicinal product.

As an advanced therapy medicinal product (ATMP), the freshly cultured allogeneic ADSCs are derived from the lipoaspirate of voluntary and informed healthy donors. ADSCs are provided according to the flowchart shown in Figure 2 by two distinct ATMP manufacturing units (EFS Auvergne Rhône Alpes, Grenoble/St-Ismier, France, and EFS Bourgogne Franche-Comté, Besançon, France) certified by EU competent authorities.

Freshly cultured allogeneic ADSCs are produced in a 1-week step, from a full-qualified working cell stock (WCS) issued from a unique healthy donor of adipose tissue.

A single IV infusion (placebo or ADSCs) is administered over 1 h (5 mL/min) in the stroke unit. During the infusion, the bag is regularly mixed to maintain the cells in suspension. Placebo corresponds to the vehicle media: glucose, human serum albumin, and ringer lactate. It is delivered by each ATMP manufacturing unit into an overwrapped packaging similar to that of the cell suspension. Patients, investigators, medical, nursing, and physiotherapy staff are blinded to the treatment assignment.

### 2.4 Sample size calculation

Assuming a mean NIHSS motor subscore of 3.5 in the control group and a common standard deviation of 2, a sample size of 39 patients per group achieves 90% power to reject the null hypothesis of equal means between the two groups, with a significance level (alpha) of 0.05 using a two-sided two-sample equal-variance *t*-test. We propose to include 40 patients per group in case technical issues occur with treatment delivery.

Eighty patients (40 in the placebo group and 40 in the treatment group) will be enrolled. We plan to include one patient per month per center, based on the inclusion criteria and the number of patients admitted to our stroke centers.

### 2.5 Randomization procedures

The randomization sequence has been generated using a computer before the study implementation. We use a dynamic allocation method based on age, severity (NIHSS score), side of infarction, recanalization procedure (thrombectomy and/or thrombolysis), and center. We randomize patients in a 1:1 ratio to receive an IV infusion of placebo or 3 million ADSC/kg within 10 days after stroke onset.

### 2.6 Follow-up

As shown in Figure 1, follow-up visits are scheduled at 2 weeks, 3 months, 6 months, 1 year, and 2 years following stroke to assess clinical scores and collect standard blood tests. Rehabilitation measures are assessed at 2 weeks, 6 months, and 1 year by a physiotherapist to independently assess patients' sensorimotor recovery. A multimodal MRI is performed at baseline and 6 months following stroke for safety and efficacy assessment.

### 2.7 Primary outcome

The primary efficacy outcome is the motor sub-score of the NIHSS, computed as the sum of the upper limb, lower limb, and hand scores, measured over time from baseline to 6 months visits in the ADSC group compared to the placebo group.

### 2.8 Secondary outcomes

*Second*ary outcomes include the following:

- Safety outcomes: This will involve monitoring for mortality and adverse events, specifically focusing on allergic reactions occurring within the first 24 h following treatment infusion, clinical perturbations (e.g., recurrent stroke and thromboembolic disease) occurring within the first week following infusion, and blood test abnormalities (e.g., hepatic cytolysis and leukocytosis). Additionally, MRI changes on T_1_ and Fluid-attenuated inversion recovery (FLAIR) images will be assessed, along with immunomonitoring for cross-reaction between host and allogeneic products and the presence of anti-Human Leukocyte Antigen (HLA) antibodies.
- Behavioral measures using clinical and rehabilitation scores are to be collected from baseline to the 2-year follow-up visit. Clinical measures include the NIHSS, detailing each item, including a “hand motricity” assessment; a modified Rankin Scale (mRS) score, an Aphasia Handicap Score, a Montreal Cognitive Assessment score, a Depression Intensity Scale Circles score, a Fatigue Scale score, a Work and Social Adjustment Scale score, a Euro-Quality of Life 5-level visual analogue scale (EuroQol EQ5 VAS) score, and a 10 m walking test score. Rehabilitation measures include the motor part of the Fugl–Meyer score to assess sensorimotor recovery, the Postural Assessment for Stroke Scale for postural control evaluation, and the Ashworth Scale for spasticity assessment.
- The brain MRI protocol includes T_1_-weighted and FLAIR sequences to assess safety, stroke lesion side and volume, and cortical thickness; diffusion sequences to assess white matter micro-structural alterations; and motor task-related functional MRI activity to assess the effects of ADSC compared to placebo from baseline to 6-month follow-up (Jaillard et al., 2020). Additional optional MRI sequences include magnetic resonance angiography (MRA) and arterial spin labeling perfusion; multi-parameter quantitative brain MRI maps to assess axon, myelin, iron, and water concentration of lesioned brain tissue; and exploratory magnetic resonance (MR) fingerprinting sequence to compute relaxometry maps, cerebral blood volume, and brain oxygenation maps (Christen et al., 2014). MRI biomarkers will also be used to improve our understanding of brain repair mechanisms and the effects of ADSC therapy on post-stroke brain remodeling.
- An immunomonitoring study is performed in the 30 first patients at baseline and 3-month follow-up to assess safety and explore the immunomodulation effect of ADSCs.

### 2.9 Statistical analysis

All primary and secondary outcomes will be analyzed in the intent-to-treat population. Additional analysis will be performed using a modified analysis set (all patients who are randomized and still alive at 6 months + 1-week post-randomization) and in the per-protocol population (all randomized patients who received the complete assigned study treatment and complied with all inclusion and non-inclusion criteria). A safety analysis will be performed on the whole study population, comparing all randomized patients who received any amount of the planned ADSC treatment to patients who received a placebo.

The primary effectiveness outcome is the change in the NIHSS motor sub-score at 6 months. The change in the NIHSS motor sub-score is a quantitative value and will be analyzed using a mixed model for repeated measures including patients as a random effect and as fixed effects: baseline NIHSS motor score, visit, treatment, and interaction between treatment and visit. Gender and stratification variables for randomization (age, recanalization procedure, lesion side, and center) in the model will be adjusted.

Comparisons between treated and control groups will be conducted using Student's *t*-test or the Mann–Whitney *U* test for continuous outcomes, the chi-squared test or Fisher's exact test for binary outcomes, and the Mantel–Haenszel chi-squared test or the Cochran–Mantel–Haenszel test for ordinal outcomes.

The treatment effect on the change from baseline for secondary efficacy behavioral outcomes will be tested, as for the primary outcome, using mixed models for repeated measures. At the 2-year follow-up visit, the mean change from baseline between treatment groups will be compared to the minimum clinically important difference for each score, provided that such data are available. An alternative analysis will categorize scores using published or specific cutoff scores, which will be reported as part of the study results.

A shift analysis of the mRS scores will be considered using an ordinal logistic regression.

For MRI biomarkers, the same primary analysis of treatment effect on change from baseline will be performed.

To account for multiple tests, a false discovery rate approach will be used to control the proportion of false hypotheses rejected for MRI data or blood biomarkers.

Safety outcomes occurring in the first 24 h and during the study will be described by the treatment group and compared using summary statistics. The analysis will utilize various modeling frameworks, including logistic regression for event occurrence, time-to-event analysis, and count data regressions for recurrence or multiple events.

Multivariate statistical techniques (factor analysis, cluster analysis, and discriminant analysis) or more advanced methods will be performed on the data collected for the 30 first patients at baseline and the 3-month follow-up to identify the effect of treatment on the immunomonitoring study.

Unless stated, if the *p*-values for multiple tests are not adjusted, 95% confidence intervals will be used as a measure of precision. All statistical analysis will be performed using SAS (SAS Institute Inc., Cary, NC, USA), or R software (R foundation, Vienna, Austria).

### 2.10 Monitoring

The DSMB will evaluate the safety of RESSTORE. A DSMB meeting may be requested by DSMB members, the sponsor, the sponsor's safety desk, or the study's principal investigator at any time to discuss safety concerns. The DSMB will hold a meeting for safety based on case report form data and case reports of serious adverse events after 15, 30, and 60 patients have completed their 2-week follow-up.

## 3 Discussion

Regenerative therapies hold great promise for stroke treatment as they enhance several processes to promote neural repair in post-stroke recovery (Detante et al., 2023). RESSTORE will assess the effects of cell therapy using a single IV infusion of freshly cultured allogeneic ADSCs on recovery in patients with a subacute stroke. An advantage of cell therapy relates to the safety of MSC documented in several diseases and more recently in stroke (Hess et al., 2017; Moniche et al., 2023; Houkin et al., 2024; Jaillard et al., 2020). In acute-subacute stroke, our clinical experience corroborates the good safety of ADSC therapy (preliminary data) (Detante et al., 2022). Moreover, cell therapy can be used across a wide therapeutic time window, allowing more patients to be treated.

Regarding the efficacy of ADSC therapy, it is crucial to address some key issues: the existence of a dose–effect relationship, the timing of therapeutic effects, and the identification of responders to cell therapy. Using quantitative motor behavior assessments and multimodal MRI measurements can provide objective, precise, and accurate outcome measures. This approach may enhance the sensitivity in detecting treatment effects and help identify responders to cell therapy.

The original aspect of this study is that we use freshly cultured ADSCs (not immediately injected after thawing), and complementary motor and global behavior scales coupled with advanced MRI neuromarkers that may improve our understanding of ADSC therapy on post-stroke brain remodeling. Our results will provide some insight into the design of future larger regenerative therapy trials.
