# Supplementary material for: Regenerative stem cell therapy for stroke in Europe (RESSTORE): a multicenter randomized controlled efficacy clinical trial
Source: Front Stroke. 2024 Sep 27;3:1416490. doi: 10.3389/fstro.2024.1416490 (PMC12802603; doi:10.3389/fstro.2024.1416490)
Supplement: Supplementary file 1 [file Table_1.DOCX]

Supplemental data

Regenerative Stem Cell Therapy for Stroke in Europe (RESSTORE): a multicenter randomized controlled efficacy clinical trial.

L. Legris^1^, A. Moisan^2^, A. Jaillard^3^, L. Bonnet^4^, T. Moulin^4^, I. Sibon^5^, E. Touzé^6^, I. Favre-Wiki^7^, C. Cordonnier^8^, L. Dellaschiava^8^, M. Mazighi^9^, C. Rosso^10^, S. Alamowitch^11^, D. Calvet^12^, M. Barbieux-Guillot^13^, S. Roux ^14^, A. Mojallal ^15^, F. Boucher ^15^, A. Thuriot^7^, J. Soulard^7^, B. Naegele^7^, D. Perennou^16^, M. Roustit^17^, Z. Putkaradze^17^, M. Hommel^18^, A. Lehmann^19^, J. Colombat^20^, F. Chorfa ^21^, D. Maucort-Boulch^21^, L. Lamalle^22^, S. Grand^23^, A. Krainik^24^, O. Detante^1*^

for the RESSTORE Study Group

**Figure 1.** Detailed NIHSS with motor hand item.

| 1a-Level of consciousness | 0 = Alert; keenly responsive  1 = Not alert, but arousable by minor stimulation  2 = Not alert; requires repeated stimulation  3 = Unresponsive or responds only with reflex |
| --- | --- |
| 1b-Level of consciousness questions:  “What is your age?”  “What is the month?” | 0 = Answers two questions correctly  1 = Answers one question correctly  2 = Answers neither questions correctly |
| 1c-Level of consciousness commands:  “Open and close your eyes”  “Grip and release your hand” | 0 = Performs both tasks correctly  1 = Performs one task correctly  2 = Performs neither task correct |
| 2- Best gaze | 0 = Normal  1 = Partial gaze palsy  2 = Forced deviation |
| 3-Visual | 1 = Partial hemianopia  2 = Complete hemianopia  3 = Bilateral hemianopia |
| 4-Facial palsy | 0 = Normal symmetric movements  1 = Minor paralysis  2 = Partial paralysis  3 = Complete paralysis of one or both sides |
| 5-Motor arm  Left arm  Right arm | 0 = No drift  1 = Drift  2 = Some effort against gravity  3 = No effort against gravity  4 = No movement |
| 6-Motor leg  Left leg  Right leg | 0 = No drift  1 = Drift  2 = Some effort against gravity  3 = No effort against gravity  4 = No movement |
| 7-Limb ataxia | 0 = Absent  1 = Present in one limb  2 = Present in two limbs |
| 8-Sensory | 0 = Normal; no sensory loss  1 = Mild-to-moderate sensory loss  2 = Severe-to-total sensory loss |
| 9-Best language | 0 = No aphasia; normal  1 = Mild-to-moderate aphasia  2 = Severe aphasia  3 = Mute; global aphasia |
| 10-Dysarthria | 0 = Normal  1 = Mild-to-moderate dysarthria  2 = Severe dysarthria |
| 11-Extinction and inattention | 0 = No abnormality  1 = Visual, tactile, auditory, spatial, or personal inattention  2 = Profound hemi-inattention or extinction |
| 12- Motor hand | 0 = No motor deficit  1 = Partial extension of the hand  2 = No movement |
